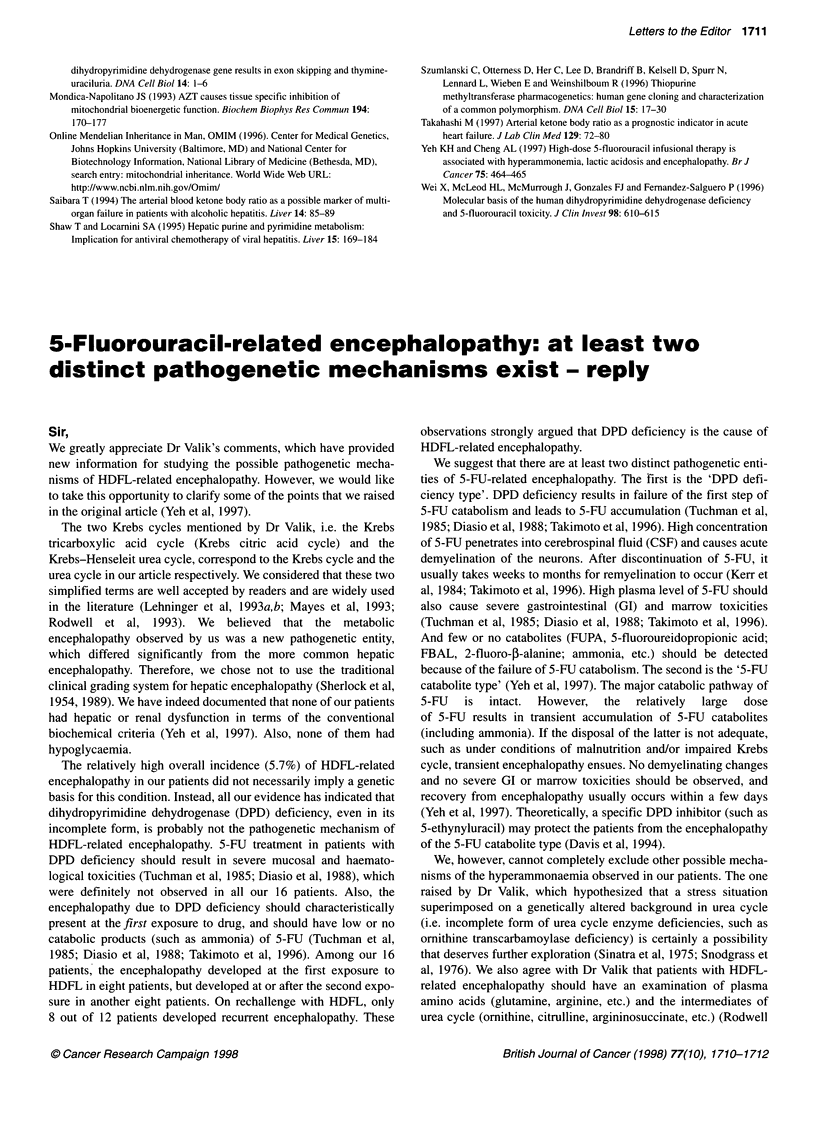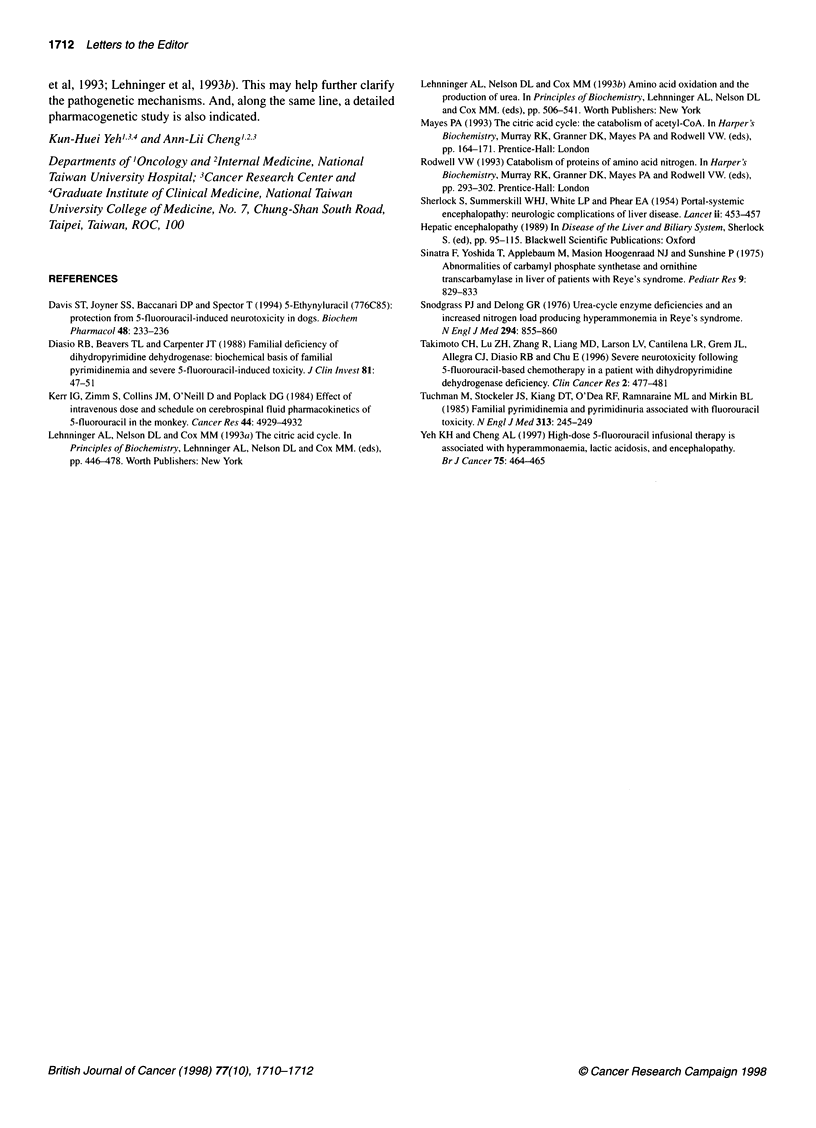# 5-Fluorouracil-related encephalopathy: at least two distinct pathogenetic mechanisms exist - reply

**Published:** 1998-05

**Authors:** Kun-Huei Yeh, Ann-Lii Cheng


### 5-Fluorouracil-related encephalopathy: at least two distinct pathogenetic mechanisms exist – reply

**Sir,**

We greatly appreciate Dr Valik's comments, which have provided new information for studying the possible pathogenetic mechanisms of HDFL-related encephalopathy. However, we would like to take this opportunity to clarify some of the points that we raised in the original article (Yeh et al, 1997).

The two Krebs cycles mentioned by Dr Valik, i.e. the Krebs tricarboxylic acid cycle (Krebs citric acid cycle) and the Krebs–Henseleit urea cycle, correspond to the Krebs cycle and the urea cycle in our article respectively. We considered that these two simplified terms are well accepted by readers and are widely used in the literature (Lehninger et al, 1993*a,b*; Mayes et al, 1993; Rodwell et al, 1993). We believed that the metabolic encephalopathy observed by us was a new pathogenetic entity, which differed significantly from the more common hepatic encephalopathy. Therefore, we chose not to use the traditional clinical grading system for hepatic encephalopathy (Sherlock et al, 1954, 1989). We have indeed documented that none of our patients had hepatic or renal dysfunction in terms of the conventional biochemical criteria (Yeh et al, 1997). Also, none of them had hypoglycaemia.

The relatively high overall incidence (5.7%) of HDFL-related encephalopathy in our patients did not necessarily imply a genetic basis for this condition. Instead, all our evidence has indicated that dihydropyrimidine dehydrogenase (DPD) deficiency, even in its incomplete form, is probably not the pathogenetic mechanism of HDFL-related encephalopathy. 5-FU treatment in patients with DPD deficiency should result in severe mucosal and haematological toxicities (Tuchman et al, 1985; Diasio et al, 1988), which were definitely not observed in all our 16 patients. Also, the encephalopathy due to DPD deficiency should characteristically present at the *first* exposure to drug, and should have low or no catabolic products (such as ammonia) of 5-FU (Tuchman et al, 1985; Diasio et al, 1988; Takimoto et al, 1996). Among our 16 patients, the encephalopathy developed at the first exposure to HDFL in eight patients, but developed at or after the second exposure in another eight patients. On rechallenge with HDFL, only 8 out of 12 patients developed recurrent encephalopathy. These observations strongly argued that DPD deficiency is the cause of HDFL-related encephalopathy.

We suggest that there are at least two distinct pathogenetic entities of 5-FU-related encephalopathy. The first is the 'DPD deficiency type'. DPD deficiency results in failure of the first step of 5-FU catabolism and leads to 5-FU accumulation (Tuchman et al, 1985; Diasio et al, 1988; Takimoto et al, 1996). High concentration of 5-FU penetrates into cerebrospinal fluid (CSF) and causes acute demyelination of the neurons. After discontinuation of 5-FU, it usually takes weeks to months for remyelination to occur (Kerr et al, 1984; Takimoto et al, 1996). High plasma level of 5-FU should also cause severe gastrointestinal (GI) and marrow toxicities (Tuchman et al, 1985; Diasio et al, 1988; Takimoto et al, 1996). And few or no catabolites (FUPA, 5-fluoroureidopropionic acid; FBAL, 2-fluoro-β-alanine; ammonia, etc.) should be detected because of the failure of 5-FU catabolism. The second is the '5-FU catabolite type' (Yeh et al, 1997). The major catabolic pathway of 5-FU is intact. However, the relatively large dose of 5-FU results in transient accumulation of 5-FU catabolites (including ammonia). If the disposal of the latter is not adequate, such as under conditions of malnutrition and/or impaired Krebs cycle, transient encephalopathy ensues. No demyelinating changes and no severe GI or marrow toxicities should be observed, and recovery from encephalopathy usually occurs within a few days (Yeh et al, 1997). Theoretically, a specific DPD inhibitor (such as 5-ethynyluracil) may protect the patients from the encephalopathy of the 5-FU catabolite type (Davis et al, 1994).

We, however, cannot completely exclude other possible mechanisms of the hyperammonaemia observed in our patients. The one raised by Dr Valik, which hypothesized that a stress situation superimposed on a genetically altered background in urea cycle (i.e. incomplete form of urea cycle enzyme deficiencies, such as ornithine transcarbamoylase deficiency) is certainly a possibility that deserves further exploration (Sinatra et al, 1975; Snodgrass et al, 1976). We also agree with Dr Valik that patients with HDFL-related encephalopathy should have an examination of plasma amino acids (glutamine, arginine, etc.) and the intermediates of urea cycle (ornithine, citrulline, argininosuccinate, etc.) (Rodwell

© Cancer Research Campaign 1998 

et al, 1993; Lehninger et al, 1993*b*). This may help further clarify the pathogenetic mechanisms. And, along the same line, a detailed pharmacogenetic study is also indicated.

*Kun-Huei Yeh*[1,3,4] *and Ann-Lii Cheng*[1,2,3]

*Departments of* [1]*Oncology and* [2]*Internal Medicine, National Taiwan University Hospital;* [3]*Cancer Research Center and* [4]*Graduate Institute of Clinical Medicine, National Taiwan University College of Medicine, No. 7, Chung-Shan South Road, Taipei, Taiwan, ROC, 100*

### REFERENCES

Davis ST, Joyner SS, Baccanari DP and Spector T (1994) 5-Ethynyluracil (776C85): protection from 5-fluorouracil-induced neurotoxicity in dogs. *Biochem Pharmacol* **48**: 233–236

Diasio RB, Beavers TL and Carpenter JT (1988) Familial deficiency of dihydropyrimidine dehydrogenase: biochemical basis of familial pyrimidinemia and severe 5-fluorouracil-induced toxicity. *J Clin Invest* **81**: 47–51

Kerr IG, Zimm S, Collins JM, O'Neill D and Poplack DG (1984) Effect of intravenous dose and schedule on cerebrospinal fluid pharmacokinetics of 5-fluorouracil in the monkey. *Cancer Res* **44**: 4929–4932

Lehnninger AL, Nelson DL and Cox MM (1993*a*) The citric acid cycle. In *Principles of Biochemistry*, Lehnninger AL, Nelson DL and Cox MM. (eds), pp. 446–478. Worth Publishers: New York

Lehnninger AL, Nelson DL and Cox MM (1993*b*) Amino acid oxidation and the production of urea. In *Principles of Biochemistry*, Lehnninger AL, Nelson DL and Cox MM. (eds), pp. 506–541. Worth Publishers: New York

Mayes PA (1993) The citric acid cycle: the catabolism of acetyl-CoA. In *Harper's Biochemistry*, Murray RK, Granner DK, Mayes PA and Rodwell VW. (eds), pp. 164–171. Prentice-Hall: London

Rodwell VW (1993) Catabolism of proteins of amino acid nitrogen. In *Harper's Biochemistry*, Murray RK, Granner DK, Mayes PA and Rodwell VW. (eds), pp. 293–302. Prentice-Hall: London

Sherlock S, Summerskill WHJ, White LP and Phear EA (1954) Portal-systemic encephalopathy: neurologic complications of liver disease. *Lancet* **ii**: 453–457

Hepatic encephalopathy (1989) In *Disease of the Liver and Biliary System*, Sherlock S. (ed), pp. 95–115. Blackwell Scientific Publications: Oxford

Sinatra F, Yoshida T, Applebaum M, Masion Hoogenraad NJ and Sunshine P (1975) Abnormalities of carbamyl phosphate synthetase and ornithine transcarbamylase in liver of patients with Reye's syndrome. *Pediatr Res* **9**: 829–833

Snodgrass PJ and Delong GR (1976) Urea-cycle enzyme deficiencies and an increased nitrogen load producing hyperammonemia in Reye's syndrome. *N Engl J Med* **294**: 855–860

Takimoto CH, Lu ZH, Zhang R, Liang MD, Larson LV, Cantilena LR, Grem JL, Allegra CJ, Diasio RB and Chu E (1996) Severe neurotoxicity following 5-fluorouracil-based chemotherapy in a patient with dihydropyrimidine dehydrogenase deficiency. *Clin Cancer Res* **2**: 477–481

Tuchman M, Stockeler JS, Kiang DT, O'Dea RF, Ramnaraine ML and Mirkin BL (1985) Familial pyrimidinemia and pyrimidinuria associated with fluorouracil toxicity. *N Engl J Med* **313**: 245–249

Yeh KH and Cheng AL (1997) High-dose 5-fluorouracil infusional therapy is associated with hyperammonaemia, lactic acidosis, and encephalopathy. *Br J Cancer* **75**: 464–465

© Cancer Research Campaign 1998